# Detection of Age-Related Cognitive Decline Over Short Time Frames Using Digital Multiday Learning Assessments: Longitudinal Study

**DOI:** 10.2196/100917

**Published:** 2026-10-27

**Authors:** Iris Blotenberg, Hairin Kim, Daniel Soberanes, Mark A Dubbelman, Lex R Minto, Onyinye J Udeogu, Vivienne Lubbat, Rachel F Buckley, Gad A Marshall, Keith A Johnson, Dorene M Rentz, Reisa A Sperling, Kathryn V Papp, Rebecca E Amariglio

**Affiliations:** 1 German Center for Neurodegenerative Diseases (DZNE), Greifswald, Germany; 2 Department of Neurology, Mass General Brigham, Harvard Medical School, 60 Fenwood Road, Boston, MA, United States, 1 617-732-8060; 3 Melbourne School of Psychological Sciences, University of Melbourne, Melbourne, VIC, Australia; 4 Department of Radiology, Mass General Brigham, Harvard Medical School, Boston, MA, United States

**Keywords:** cognitive aging, older adults, digital cognitive assessment, remote assessment, response time, computational modeling, longitudinal study, associative memory, learning, mobile health, digital biomarkers, Alzheimer disease

## Abstract

**Background:**

High-resolution digital cognitive tools administered at frequent intervals enable the study of age-related cognitive change at a previously unattainable level of granularity. Computational modeling further enables the simultaneous examination of multiple aspects of cognitive performance.

**Objective:**

The objective of this study was to examine whether repeated digital multiday learning assessments can detect age-related cognitive decline over short time frames in cognitively unimpaired older adults and to quantify age-related differences in process-based cognitive markers derived from computational modeling.

**Methods:**

A total of 209 cognitively unimpaired older adults (mean age 73.6, SD 8.2 years; n=136, 65.1% female) from the Harvard Aging Brain Study and affiliated studies were administered the Boston Remote Assessment for Neurocognitive Health, which includes the face-name task, a paired associative learning task. Daily median response times for correct responses were used as the primary outcome. A new version of the face-name task was administered across up to 4 testing intervals over a mean of 2.8 (SD 0.4) years. Using computational modeling, we estimated longitudinal changes in peak performance, a practice-adjusted marker of an individual’s best expected response time, as an indicator of age-related cognitive decline. We further examined age-related differences in peak performance, stability (within-person variability), learning within and across testing intervals, and between-interval forgetting, with embedded linear regressions for age, sex, years of education, and device type.

**Results:**

Peak performance slowed by 0.04 seconds per year (95% credible interval 0.02-0.06; approximately 2% per year), indicating age-related cognitive decline. Higher age (+1-SD) was associated with slower peak performance (+0.19 seconds, 95% credible interval 0.12-0.26), steeper annual slowing in peak performance (+0.036 seconds per year), greater within-person variability (+0.043 seconds), reduced learning across testing intervals, and more forgetting.

**Conclusions:**

Our findings indicate that a digital multiday learning paradigm combined with computational modeling of response times sensitively detects age-related cognitive decline over short timescales (<3 years), which are difficult to capture in conventional study designs relying on infrequent assessments. In addition, this approach identifies specific process-based cognitive markers associated with aging, including slower performance, reduced stability, and diminished learning across testing intervals.

## Introduction

Even in the absence of Alzheimer disease or other neurodegenerative disorders, older adults experience gradual cognitive decline, particularly in fluid abilities such as processing speed and episodic memory—a phenomenon commonly referred to as normative cognitive aging [1,2]. These age-related changes manifest not only in reduced task accuracy but also in decreased efficiency and stability of underlying cognitive processes. Core markers of cognitive aging include slower and more variable performance as well as reduced capacity to learn and retain new information [3-6]. Such process-level changes can be sensitively captured using response times, which provide fine-grained, temporally resolved measures of cognitive function and allow for the assessment of the efficiency and stability of underlying cognitive processes [7], extending information beyond accuracy-based measures.

A precise characterization of cognitive aging in cognitively unimpaired older adults is essential as it provides an important reference for distinguishing age-related change from that of pathological trajectories such as Alzheimer disease. In many cognitive aging cohorts, biomarker data are unavailable, making it difficult to determine whether observed longitudinal changes may be partly attributable to early preclinical Alzheimer disease pathology [8]. As a result, it remains unclear whether age-related cognitive decline can be reliably detected over short timescales in cognitively unimpaired older adults and to what extent distinct cognitive processes—such as speed and stability of performance, as well as learning and retention—are differentially affected by age.

Addressing this question—particularly detecting gradual cognitive change over relatively short timescales—is methodologically challenging when using traditional longitudinal study designs [9-12]. These studies typically rely on infrequent, single-administration cognitive assessments administered years apart and focus on global accuracy or mean response time scores [13]. As a consequence, short-term fluctuations due to sleep, mood, or environmental factors may obscure long-term cognitive change. At the same time, repeated testing introduces substantial practice effects, which may equal or exceed true age-related decline, especially over brief follow-up intervals of only a few years [3,9,14,15]. Consequently, observed changes in test scores reflect a composite of practice-related gains, day-to-day variability, and underlying cognitive decline, limiting interpretability and reducing sensitivity to individual differences.

Recent advances in digital cognitive assessment [16,17] provide a promising avenue for addressing these challenges. Remote cognitive testing conducted repeatedly over multiple days enables the capture of cognitive processes with high temporal resolution. In parallel, computational modeling approaches applied to intensive longitudinal response time data allow practice-related gains to be separated from cognitive change and enable the decomposition of performance into theoretically meaningful process-based markers, including speed and stability of performance as well as learning rate and forgetting [14,15]. A key advantage of this approach is that change in peak performance across testing intervals can be interpreted as a practice-adjusted estimate of within-person cognitive change over time. This approach leverages the fine-grained temporal information provided by response time data to characterize the efficiency and stability of underlying cognitive processes, complementing information obtained from accuracy-based measures [7].

In the present study, we leveraged a multiday associative face-name memory task from the Boston Remote Assessment for Neurocognitive Health (BRANCH) battery [18,19] administered daily over 7 consecutive days and repeated approximately every 6 months.

Our aims were twofold: (1) to evaluate whether this paradigm detects age-related cognitive decline over short time frames in cognitively unimpaired older adults and (2) to estimate the direction and magnitude of age associations with latent process-based cognitive markers.

## Methods

### Participants

Participants were recruited from 3 Mass General Brigham observational cohort studies: the Harvard Aging Brain Study (2P01AG036694-11; principal investigators: RAS and KAJ), the Instrumental Activities of Daily Living study (R01AG053184 and R01AG067021; principal investigator: GAM), and the Subjective Cognitive Decline study (R01AG058825-01A; principal investigator: REA). Participants were community dwelling and required to remain cognitively unimpaired throughout the study period. Cognitive status was assessed at annual in-clinic visits and was defined based on a Clinical Dementia Rating global score of 0, a Mini-Mental State Examination score greater than 25, and a logical memory delayed recall score above education-adjusted thresholds (>4 for 8-15 years of education; >8 for ≥16 years of education). Only participants who met these criteria at every available clinical assessment during follow-up were included. The neuropsychological assessment closest to the first BRANCH testing interval was conducted, on average, 29.7 (SD 121) days before BRANCH testing. The corresponding mean time differences were 98.8 (SD 135) days for the second interval, 202.0 (SD 149) days for the third interval, and 196.0 (SD 183) days for the fourth interval. Exclusion criteria included a primary psychiatric diagnosis, unstable medical conditions, severe head trauma, and a recent history of alcohol or substance abuse.

### Materials and Procedure

Digital cognitive assessment was conducted using BRANCH, a previously validated web-based platform for remote cognitive testing that includes 2 associative memory tasks and 1 processing speed task [20]. Participants completed the tests independently and remotely on a daily basis over 7 consecutive days, enabling the characterization of learning dynamics.

The present study focused on the Face-Name Test, an associative memory task, which is illustrated in Figure 1. Participants were instructed to learn 20 face-name pairs. Following a 5-minute delay, they completed two tasks: (1) *first-letter name recall* (FNLT), in which they identified the first letter of the associated name for each face; and (2) *face-name matching* (FNMT), in which they selected the correct name from 3 multiple-choice options. The primary outcome of interest in the present study was response time for correct responses, which was recorded at the item level. First, median response times for correctly answered items were calculated separately for FNLT and FNMT. These task-specific median response times were then averaged to ensure equal weighting of both tasks. Across testing days, daily response time estimates were based on an average of 28.7 (SD 10.4) correctly answered trials across both subtasks out of 40 possible trials per testing day.

**Figure 1.**
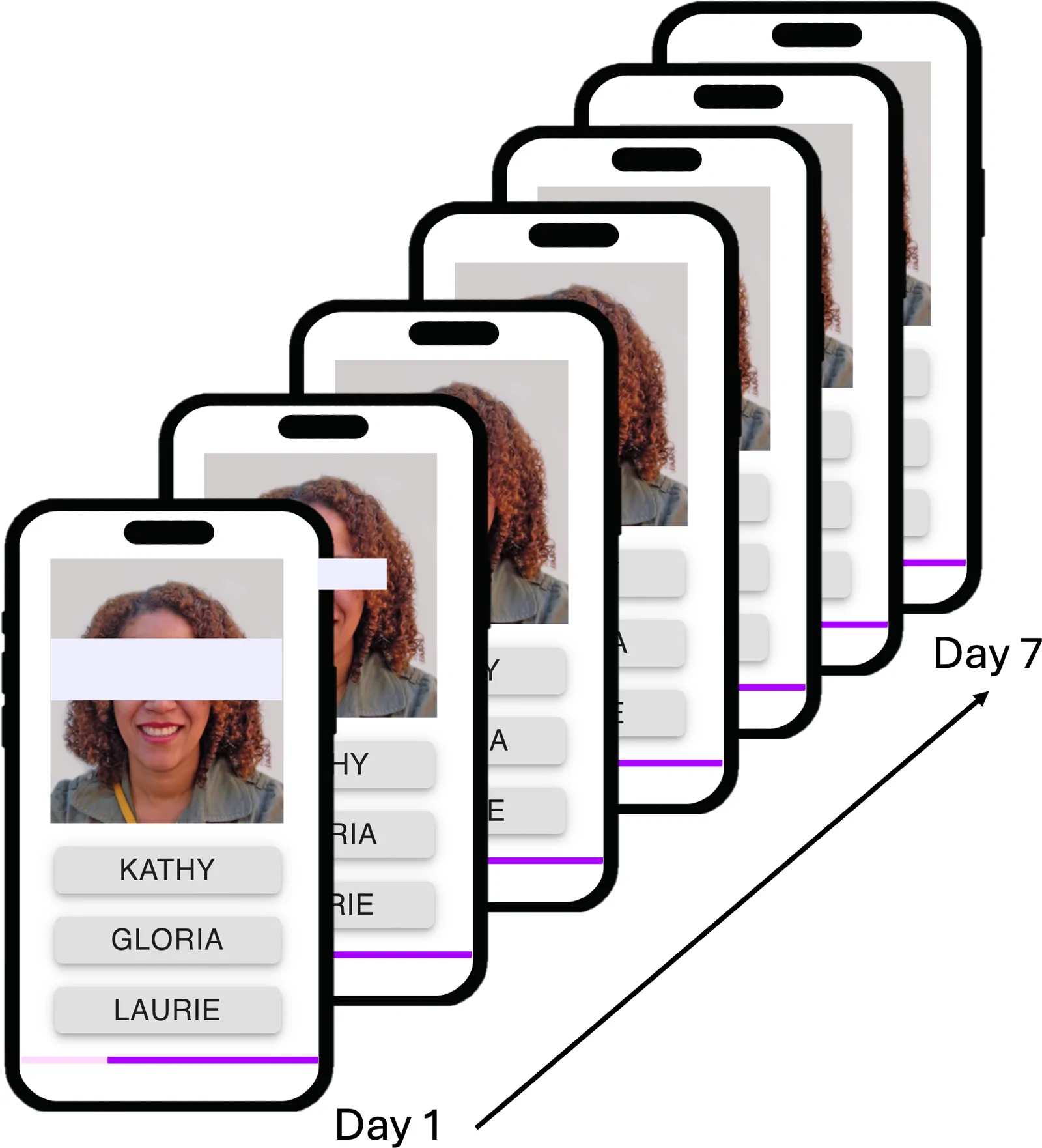
Illustration of the face-name matching task. Although written consent for publication was obtained, the facial image has been blurred in accordance with journal policy to protect participant privacy.

Participants completed up to 4 multiday BRANCH testing intervals. Each interval used the same face-name paradigm but a different set of face-name pairs, thereby serving as an alternate-form version of the task. Alternate forms were developed according to prespecified stimulus construction rules to maintain consistency in task structure and difficulty. In an independent pilot study, the first 3 forms were administered in counterbalanced order and showed broadly comparable accuracy and modest differences in response time, with no systematic increase in response time across forms. Further details are provided in Multimedia Appendix 1. Within each interval, the same stimulus set was administered repeatedly over 7 consecutive days to characterize multiday learning. Testing intervals were designed to include 7 consecutive testing days; participants contributed an average of 6.5 (SD 1.2) testing days per interval. The second testing interval followed an average of 1.2 (SD 0.4) years after the first testing interval, the third followed an average of 2.2 (SD 0.6) years after, and the fourth followed an average of 2.8 (SD 0.4) years after.

### Statistical Analysis

We used a computational phenotyping approach to model daily median response times from correct trials across repeated 7-day testing intervals administered over multiple years. No additional response time trimming or exclusion procedures were applied before calculating the daily median. Specifically, we estimated a Bayesian double-negative exponential model (BDEM) proposed by Oravecz et al [14,15]. Full model specification and estimation details are provided in Multimedia Appendix 1.

Conceptually, the model assumes that observed performance reflects several processes occurring simultaneously: short-term learning across days within a testing interval, longitudinal practice-related gains across repeated testing intervals, forgetting of prior task-related practice gains between intervals, and longer-term change in peak performance. The 2 exponential components of the model capture learning on different timescales: one models rapid learning within each 7-day testing interval, whereas the other models slower practice-related gains that accumulate across repeated testing intervals and reflect increasing task familiarity. Within each testing interval, performance is modeled as approaching an individual’s peak performance. By modeling learning separately, change in peak performance over time can be interpreted as a practice-adjusted indicator of within-person cognitive change. Figure 2 provides a conceptual illustration of the model and the process-based cognitive markers derived from it.

**Figure 2.**
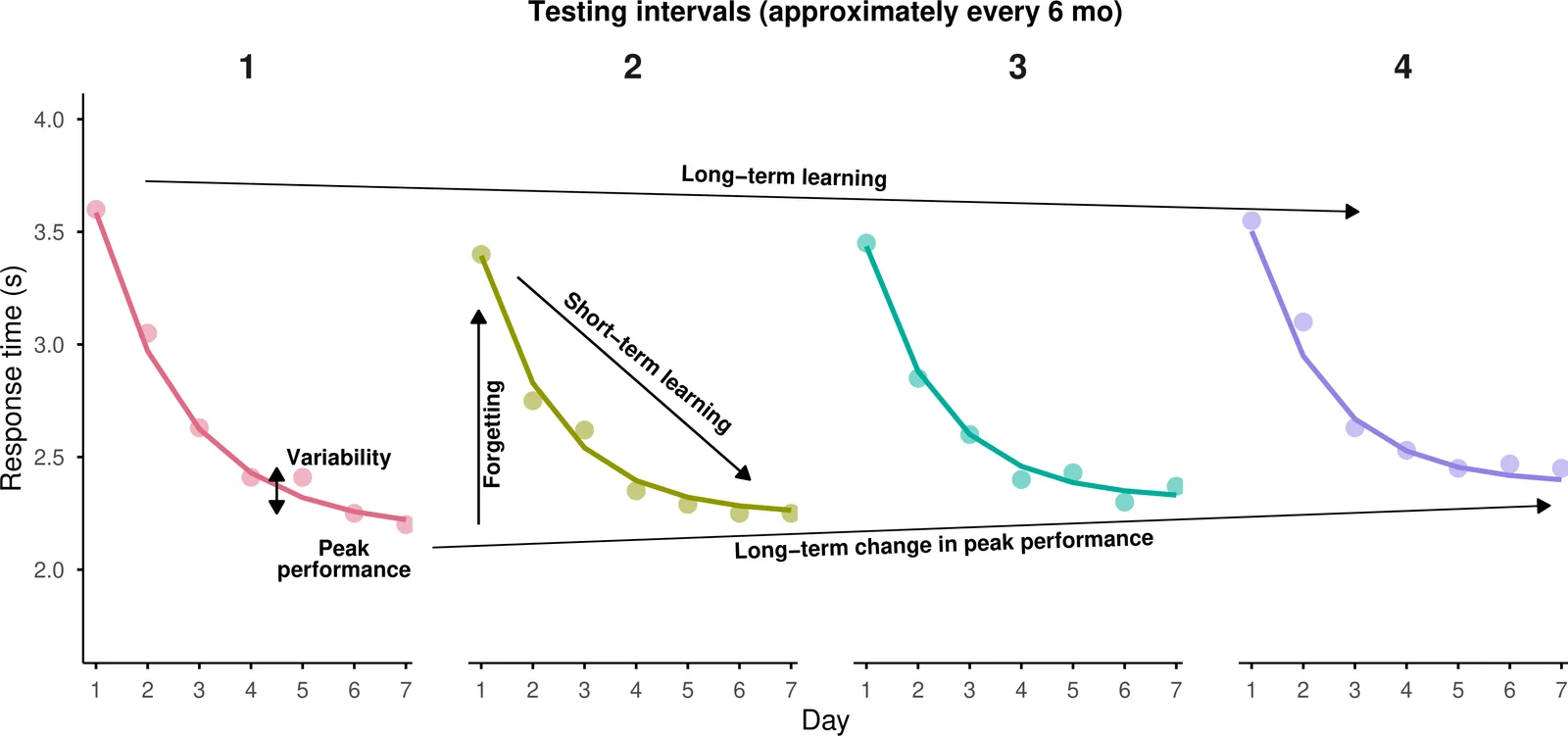
Illustration of the Bayesian double-negative exponential model with 4 testing intervals for 1 synthetic participant. Data are displayed as dots. Corresponding negative exponential curves (solid lines) show model fit.

*Peak performance* represents an individual’s estimated best expected response time within a testing interval, corresponding to the asymptote approached during short-term learning. *Change in peak performance* across testing intervals reflects longer-term, practice-adjusted within-person cognitive change and is interpreted as an indicator of cognitive aging. *Short-term learning rate* quantifies how quickly individuals approach their peak performance across repeated exposure within a 7-day testing interval. In contrast, *long-term learning rate* captures the rate of task familiarization that accumulates across repeated testing intervals and unfolds over a longer timescale than learning within an interval. In addition, the model estimates within-person variability, indexing performance inconsistency around the predicted trajectory, and forgetting between testing intervals, reflecting the loss of prior task-related practice gains.

To examine age effects and further potential associations between cognitive markers and sociodemographic variables, age, sex, and years of education were included as predictors, along with a control variable for the device used (computer or smartphone). Age and years of education were *z* standardized to facilitate comparison of their associations across the process-based cognitive markers. In the present sample, 1 SD corresponded to 8.2 years of age and 2.5 years of education. These regression parameters were embedded in the BDEM in a 1-step analysis such that all reported results represented partial regression coefficients, summarizing the effect of each predictor conditional on all others.

The model used all available median response times. Missing testing days and testing intervals contributed no likelihood term. Accordingly, the primary analysis assumed that missingness was conditionally independent of unobserved response times after accounting for the observed covariates and the longitudinal performance patterns captured by the model.

All analyses were conducted in R (version 4.4.1; R Foundation for Statistical Computing) [21]. Model fitting was performed in Stan using the *rstan* interface [22]. Convergence was assessed using standard diagnostics, and model adequacy was evaluated using posterior predictive checks [23].

### Sensitivity Analysis

Several additional analyses were conducted to assess the robustness of the findings and examine the time frame over which age-related cognitive change could be detected. First, to examine whether the age-related findings persisted in the absence of elevated amyloid burden, an important marker of preclinical Alzheimer disease, analyses were repeated in a subset of participants with available amyloid positron emission tomography, restricting analyses to those classified as amyloid beta–negative (n=167). Details on positron emission tomography image acquisition and processing are provided in Multimedia Appendix 1. Second, the analyses were repeated using 3 and 2 testing intervals to evaluate the timescale at which age-related cognitive changes can be detected within the present paradigm. Third, the analyses were repeated separately for FNLT and FNMT to examine whether observed effects were driven by one specific subtask. Fourth, to examine whether the findings were sensitive to selective attrition, we repeated the analysis in participants who completed the first 3 of the 4 testing intervals using only data from these 3 intervals. This restriction balanced completeness of follow-up against retention of a sufficiently large sample. Fifth, to examine whether the number of correct responses used to calculate the daily median response times varied systematically with age, we fitted a beta-binomial mixed-effects model to account for overdispersion in the daily accuracy data. Daily accuracy was modeled using the number of correct responses out of 40 trials per testing day, with age, testing interval, study day, sex, education, and device type as fixed effects and a random intercept for participant. Age and education were *z* standardized.

### Ethical Considerations

All participants provided written informed consent prior to study participation. The study protocol was approved by the Mass General Brigham Institutional Review Board (2019P000476). Data were handled in accordance with applicable data protection regulations and were stored and analyzed in deidentified form. Participants were compensated for each attempted assessment session.

## Results

### Sample Characteristics

Data from 209 participants who completed up to 4 testing intervals were analyzed (mean age at study entry 73.6, SD 8.2 years; n=136, 65.1% female). Sample characteristics are summarized in Table 1. Of these participants, 62.7% (n=131) completed the second testing interval, 51.7% (n=108) completed the third, and 34.9% (n=73) completed the fourth (Figure S1 in Multimedia Appendix 1). Baseline characteristics were broadly similar between participants who completed all 4 testing intervals and those who did not, although completion status differed by cohort source (Table S1 in Multimedia Appendix 1).

**Table 1.** Demographic characteristics (N=209).

Variables	Values
Age (y), mean (SD)	73.6 (8.2)
Sex, n (%)	
Female	136 (65.1)
Male	73 (34.9)
Race, n (%)	
Asian	5 (2.4)
Black	23 (11)
White	180 (86.1)
Other	1 (0.5)
Ethnicity, n (%)	
Hispanic	4 (1.9)
Non-Hispanic	205 (98.1)
Years of education, mean (SD)	16.5 (2.5)
MMSE^a^, mean (SD)	28.9 (1.2)
Global CDR^b^, n (%)	
0	209 (100)

a MMSE: Mini-Mental State Examination.

b CDR: Clinical Dementia Rating.

### Description of Reaction Times

Correct response counts and accuracy by testing interval and study day are provided in Table S2 in Multimedia Appendix 1. To examine whether cognitive decline was observable in the raw response time data, we calculated the average response times for each participant and testing interval. Response times initially decreased substantially from the first to the second testing interval (mean 2.63, SD 0.65 seconds in the first; mean 2.47, SD 0.61 seconds in the second) before reaching a plateau in subsequent intervals (mean 2.46, SD 0.56 seconds in the third; mean 2.46, SD 0.50 seconds in the fourth). Over a mean observation period of 2.8 (SD 0.4) years, no clear age-related slowing was evident in the raw response time data.

### BDEM

The observation-level *R*^2^ was 0.87, indicating a good descriptive fit of the BDEM to the observed face-name response time data. Group-level summaries are shown in Table 2. Within the Bayesian framework, 95% credible intervals represent the range within which a parameter value lies with 95% probability, thereby reflecting the uncertainty surrounding the estimate [23]. For each cognitive marker, 2 population-level estimates are reported: the population mean across all participants and the population SD, which reflects the extent of variability in that marker.

**Table 2.** Group-level (population) estimates of Bayesian double-negative exponential model parameters.

Group-level cognitive marker	Posterior mean (SD; 95% credible interval)^a^
Peak performance (s)	2.10 (0.37; 2.05-2.15)
Change in peak performance (s/y)	0.04 (0.07; 0.02-0.06)
Forgetting between intervals (s)	1.05 (0.44; 0.99-1.10)
Short-term learning rate^b^	0.71 (0.31; 0.65-0.79)
Long-term learning rate^b^	0.64 (0.27; 0.58-0.70)
Within-person variability (s)	0.24 (0.10; 0.24-0.25)

a This represents the posterior mean of the group-level mean and the between-person SD.

b Learning rates are exponential model parameters reflecting short-term relearning within testing intervals and cumulative practice-related learning across testing intervals.

Model-estimated peak performance, derived from daily response times across the 7-day testing intervals, was 2.10 (SD 0.37) seconds on average. Peak performance slowed by 0.04 seconds per year (95% credible interval 0.02-0.06). Relative to mean peak performance, this corresponds to an average slowing of approximately 2% per year. At the beginning of a new testing interval, performance was substantially slower, as captured in the forgetting parameter: participants started, on average, 1.05 (SD 0.44) seconds slower compared to the end of the previous testing interval. Mean learning rates within (mean 0.71, SD 0.31) and across (mean 0.64, SD 0.27) testing intervals were similar. Mean within-person variability in performance was 0.24 (SD 0.10) seconds.

Results from the embedded linear regressions are shown in Table 3. Associations with age and educational attainment are reported per 1-SD increase as both variables were *z* standardized. Chronological age was associated with multiple cognitive markers. A 1-SD higher age was associated with 0.19 seconds slower peak performance. Higher age was also associated with more pronounced annual slowing in peak performance (mean 0.036 seconds per year). Older participants further showed greater within-person variability (mean 0.043 seconds), reduced benefit from prior test exposure as reflected in greater forgetting (mean 0.136 seconds), and a lower long-term learning rate (mean –0.15). Short-term learning rate was not clearly associated with age.

Additional associations were observed with sex and educational attainment. Women showed faster peak performance in the Face-Name Test than men (mean –0.177 seconds). A 1-SD increase in years of education (equivalent to 2.5 years in the present sample) was associated with faster peak performance (mean –0.052 seconds). Moreover, individuals with more years of education exhibited less pronounced slowing of peak performance over time (mean –0.017 seconds per year), indicating less cognitive aging, as well as a higher long-term learning rate (mean 0.06).

**Table 3.** Summary of results on associations between cognitive markers and person-level predictors.^a^

Cognitive marker and predictor	Results, mean (95% credible interval)
Peak performance (s)	
Age	0.19 (0.12 to 0.26)
Sex	–0.18 (–0.31 to –0.05)
Education	–0.05 (–0.11 to –0.01)
Device	0.04 (–0.07 to 0.16)
Change in peak performance (s/y)	
Age	0.04 (0.01 to 0.06)
Sex	0.00 (–0.04 to 0.05)
Education	–0.02 (–0.03 to –0.01)
Device	0.01 (–0.02 to 0.04)
Within-person variability (s)	
Age	0.04 (0.03 to 0.06)
Sex	–0.04 (–0.07 to 0.00)
Education	–0.01 (–0.02 to 0.01)
Device	0.00 (–0.04 to 0.03)
Forgetting between intervals (s)	
Age	0.14 (0.05 to 0.22)
Sex	0.10 (–0.08 to 0.29)
Education	–0.03 (–0.10 to 0.05)
Device	–0.02 (–0.18 to 0.14)
Short-term learning rate^b^	
Age	–0.08 (–0.16 to 0.00)
Sex	0.10 (–0.07 to 0.25)
Education	–0.03 (–0.10 to 0.04)
Device	0.02 (–0.13 to 0.18)
Long-term learning rate^b^	
Age	–0.15 (–0.21 to –0.09)
Sex	0.13 (–0.02 to 0.24)
Education	0.06 (0.01 to 0.11)
Device	–0.03 (–0.14 to 0.08)

a Age and years of education coefficients are reported per 1-SD increase. One SD corresponded to 8.2 years of age and 2.5 years of education. Reference categories were male for sex and computer (vs smartphone) for device type.

b Learning rates are exponential model parameters reflecting short-term relearning within testing intervals and cumulative practice-related learning across testing intervals.

### Sensitivity Analyses

First, findings were replicated in the amyloid beta–negative subsample (n=167; Tables S3 and S4 in Multimedia Appendix 1), indicating that observed age-related effects are unlikely to be primarily driven by preclinical Alzheimer disease pathology. Second, analyses restricted to 2 and 3 testing intervals (Tables S5-S8 in Multimedia Appendix 1) yielded comparable group-level estimates and replicated the pattern of age and educational attainment effects observed in the full model, with aging-related slowing emerging from 3 testing intervals onward. Third, analyses conducted separately for the FNLT and FNMT (Tables S9-S12 in Multimedia Appendix 1) showed broadly similar patterns, including longitudinal slowing in peak performance and consistent associations between higher age and slower peak performance, greater within-person variability, and lower long-term learning. An age association with between-interval forgetting was observed for the FNLT but not for the FNMT. Fourth, the completer-only analysis restricted to participants who completed the first 3 testing intervals largely replicated the pattern observed in the primary analysis (Tables S13 and S14 in Multimedia Appendix 1). Peak performance again slowed over time, and the direction and magnitude of the associations between higher age and slower peak performance, greater slowing in peak performance, greater within-person variability, more forgetting between testing intervals, and lower long-term learning were preserved. Fifth, in the beta-binomial mixed-effects model, higher age was associated with lower accuracy (odds ratio 0.72 per 1-SD increase in age, 95% CI 0.64‐0.81; *P*<.001). Accordingly, older participants tended to contribute fewer correct trials to the calculation of the daily median response times. To examine whether the findings were robust to variation in the number of correct responses, we therefore repeated the analyses including only daily median response times based on at least 15 correct trials (Tables S15 and S16 in Multimedia Appendix 1). The resulting estimates and pattern of associations were highly similar to those observed in the primary analysis.

## Discussion

### Principal Results

In this study, we showed that a digital multiday learning paradigm combined with computational modeling of response times can sensitively detect age-related cognitive decline over relatively short timescales of less than 3 years in cognitively unimpaired older adults. Across repeated testing intervals, peak performance—a measure indexing cognitive performance after accounting for practice effects—declined by approximately 2% per year, with steeper declines observed at higher ages. In addition, higher age was associated with a broader process profile characterized by slower peak performance, greater within-person variability, lower long-term learning, and greater between-interval forgetting of prior task-related practice gains. These findings replicate and extend previous work addressing a long-standing methodological challenge in cognitive aging research: detecting gradual cognitive decline over brief intervals despite practice effects and day-to-day variability.

The modeling approach applied here was specifically developed to disentangle aging-related decline from other sources of within-person change but has thus far been used primarily in the context of processing speed or working memory tasks [9,14,15]. Our study extends this work in several important ways. First, we applied the model to an associative memory task, a cognitive domain particularly vulnerable to age-related decline. Second, the persistence of the age-related findings in an amyloid beta–negative subsample reduces the likelihood that they were primarily driven by preclinical Alzheimer disease pathology. All participants also remained cognitively unimpaired throughout the study period, as confirmed by repeated in-clinic neuropsychological assessments. Together, these findings strengthen the interpretation that the observed cognitive changes are related to aging rather than primarily attributable to preclinical Alzheimer disease, although contributions from other pathologies cannot be excluded. Finally, the availability of up to 4 repeated testing intervals over nearly 3 years allowed us to assess the minimum time frame over which age-related cognitive change becomes detectable. Systematic aging effects were evident with as few as 3 testing intervals, spanning a mean period of approximately 2.2 years.

A key advantage of the modeling approach is its ability to simultaneously estimate multiple latent, process-based markers of cognitive aging [15]. Chronological age was associated with a coherent pattern of changes across these markers. Older age was linked to slower peak performance, greater within-person variability, lower long-term learning, and greater forgetting of prior task-related practice gains. Notably, short-term learning within testing intervals showed no clear association with age. These findings suggest that cognitive aging in cognitively unimpaired older adults involves coordinated changes across multiple cognitive processes that would be overlooked in analyses based solely on global accuracy or mean response times.

Importantly, substantial interindividual differences in these process-based cognitive markers were not explained by chronological age alone. In particular, higher educational attainment was associated with faster peak performance, a higher long-term learning rate, and less pronounced slowing of peak performance over time, suggesting that educational attainment may be linked to greater resilience against age-related cognitive decline. These findings indicate that the applied paradigm and computational model may also be informative for studying factors that buffer age-related cognitive aging. Additionally, women showed faster peak performance than men, potentially reflecting task-specific advantages in associative face-name processing.

### Comparison With Prior Work

The observed annual slowing in peak performance is consistent with prior work demonstrating gradual, small but systematic age-related cognitive slowing [3,7,24], although direct comparisons are limited due to differences in tasks, outcome measures, and study designs. In particular, our findings are consistent with the processing speed theory of cognitive aging, which posits generalized slowing as a primary mechanism of cognitive decline [2,25]. This slowing was more pronounced at older ages, aligning with trajectories of cognitive aging as described in the literature [26,27]. Greater within-person variability is consistent with prior evidence linking aging to reduced cognitive control and increased neural noise [4]. Similarly, lower long-term learning and greater forgetting of prior task-related practice gains are in line with theoretical accounts of age-related inefficiencies in encoding, consolidation, and retrieval of new information [28,29]. An interesting exception was short-term learning within testing intervals, which showed no clear association with age. This finding is notable in light of prior work suggesting that reduced multiday learning—often assessed via accuracy—is sensitive to early Alzheimer disease–related changes [19], raising the possibility that different process-based cognitive markers may show differential associations with cognitive aging vs early Alzheimer disease–related change.

The associations with educational attainment are consistent with theories of cognitive reserve and with prior evidence suggesting that life course factors shape trajectories of cognitive aging [8,30-32]. Against this background, the finding that higher educational attainment was linked not only to faster peak performance but also to less pronounced slowing over time and better long-term learning suggests that process-based markers derived from intensive digital assessments may be useful for studying factors that buffer age-related cognitive decline. More broadly, such process-based cognitive markers are particularly relevant for prevention and intervention research as they may serve as more sensitive outcome measures in trials aimed at preserving or enhancing cognitive functioning in later life.

### Limitations

Several limitations should be acknowledged. First, our sample consisted predominantly of highly educated, non-Hispanic White individuals, limiting generalizability to more diverse populations. Moreover, because BRANCH requires digital literacy and sustained adherence to repeated remote testing, its applicability to individuals with less experience using digital tools remains uncertain. Second, participants were not explicitly instructed to respond as quickly as possible; therefore, individual response strategies may have contributed to variability in response times despite item time limits and automatic progression. Third, alternate task forms were administered in a fixed sequence such that form and testing interval could not be disentangled. In the counterbalanced pilot study, the first 3 forms showed broadly comparable accuracy and modest differences in response time, with no systematic increase in response time across forms. Moreover, longitudinal slowing persisted when analyses were restricted to these first 3 forms. Nevertheless, residual alternate-form effects on response time cannot be fully separated from longitudinal change in the present longitudinal sample. Fourth, participant numbers decreased across testing intervals, raising the possibility of selective attrition. Although the main pattern of findings was preserved in the completer-only analysis, attrition associated with unobserved cognitive change or other unmeasured factors related to performance cannot be excluded. Fifth, because response times were analyzed for correct trials only and higher age was associated with lower accuracy, estimates for older participants tended to be based on fewer trials. However, older participants showed both slower response times and lower accuracy, which does not suggest a conventional speed-accuracy trade-off. Moreover, the findings were consistent across the 2 task-specific analyses and remained robust when the analysis was restricted to daily median response times based on at least 15 correct trials.

### Conclusions

A digital multiday learning paradigm combined with computational modeling of response times enabled the detection of age-related cognitive decline over short time frames in cognitively unimpaired older adults. Beyond longitudinal slowing in peak performance, the approach revealed a broader pattern of aging-related differences, including slower and more variable performance, lower long-term learning, and greater forgetting of prior task-related gains. Higher educational attainment was associated with a more favorable profile across several of these markers. These findings demonstrate the potential of intensive digital assessment and computational modeling to characterize cognitive aging and support the further evaluation of process-based cognitive markers as sensitive outcomes in prevention and intervention research.

## Supplementary material

10.2196/100917 Multimedia Appendix 1 Supplementary methods describing Face–Name Test stimulus development, Bayesian model specification and evaluation, and amyloid PET procedures, along with supplementary figures, descriptive statistics, and sensitivity analyses.

## Abbreviations

BDEM: Bayesian double-negative exponential model
BRANCH: Boston Remote Assessment for Neurocognitive Health
FNLT: first-letter name recall
FNMT: face-name matching
