# Supplementary material for: Detection of Age-Related Cognitive Decline Over Short Time Frames Using Digital Multiday Learning Assessments: Longitudinal Study
Source: JMIR Aging. 2026 Oct 27;9:e100917. doi: 10.2196/100917 (PMC13717328; doi:10.2196/100917)
Supplement: Supplementary file 1 [file aging-v9-e100917-s001.docx]

# Supplementary Methods

## 1. Development of the stimuli for the Face-Name task versions

Each alternate form of the Face-Name Test included 20 target faces selected according to prespecified demographic and visual criteria. The racial and ethnic composition was intended to broadly reflect the population of the target region; for the Boston-based development context, approximately 30% of target faces represented underrepresented racial and ethnic groups. Faces were shown frontally at shoulder height against a clear background. Target sets included equal numbers of male and female faces, with 20% younger-, 40% middle-aged-, and 40% older-appearing adults. The equal sex distribution was maintained within the age groups.

Forty names were selected for each alternate form, comprising 20 target names and 20 distractor names. Names were drawn from lists of commonly used names in the target region across birth decades to support familiarity and were not reused across alternate forms. For each item, three name options were constructed: the target name, a foil name assigned as the target name of another item, and a distractor name. Eight of the 20 name-option sets followed prespecified phonological or orthographic similarity rules. For one male and one female set each, the target and distractor names rhymed, the target and distractor shared the same initial letter, the foil and distractor shared the same initial letter, or the target and foil shared the same initial letter. The remaining 12 sets were constructed to avoid these features. Stimulus order and the positions of target, foil, and distractor response options were systematically varied to reduce learning based on item sequence or response position. Three presentation orders were created and repeated across the seven testing days in the sequence 1–2–3–1–2–3–1. These procedures were designed to promote comparability across alternate forms and to reduce systematic differences arising from stimulus composition, response-option structure, item order, or response position.

The first three alternate forms were evaluated in an independent counterbalanced pilot study of 38 cognitively unimpaired adults (mean age = 61 years, SD = 7.0, range = 51–76; 68% female). Face–Name Test performance was descriptively similar across the three forms. Mean Face–Name Matching accuracy was 0.699 (SD = 0.157) for Form A, 0.697 (SD = 0.174) for Form B, and 0.684 (SD = 0.175) for Form C. Mean First-Letter Name Recall accuracy was 0.334 (SD = 0.192), 0.350 (SD = 0.182), and 0.326 (SD = 0.189), respectively. Thus, the largest observed mean difference between forms was 1.5 percentage points for matching accuracy and 2.4 percentage points for first-letter recall accuracy. Response times also showed relatively modest differences across forms. Mean response times for Face–Name Matching were 2.406 s (SD = 0.342), 2.494 s (SD = 0.429), and 2.356 s (SD = 0.307) for Forms A, B, and C, respectively. Corresponding mean response times for First-Letter Recall were 3.683 s (SD = 0.846), 3.837 s (SD = 0.864), and 3.609 s (SD = 0.741). Response times did not increase monotonically across forms: Form B showed somewhat slower response times than Form A, whereas Form C showed somewhat faster response times than Form A for both subtasks. These descriptive findings do not indicate progressively increasing response-time demands across the first three alternate forms, although residual alternate-form differences cannot be excluded.

## 2. Bayesian double negative exponential model

### 2.1 Model specification

We applied the Bayesian double negative exponential model developed by Oravecz et al. (1, 2) to the BRANCH Face–Name task data. The study included up to four seven-day testing intervals, during which participants completed the task on consecutive days. The model structure and forgetting reparameterization followed the original implementation. Mathematically, the model is specified as:

Here, *RT_ti_* denotes person *i*’s response time at measurement occasion *t*. The parameter *a_i_* denotes person *i*’s asymptotic or peak performance, and *∆_i_* captures the person-specific linear change in peak performance across testing intervals, with *B_ti_* representing the start time of the testing interval in years since the first interval. This change in peak performance is modeled separately from learning-related processes through the two exponential functions in the equation. The first exponential function captures learning across the study, with *M_ti_* indexing cumulative measurement occasions across testing intervals, *ri* the person-specific learning rate across the study, and *gi* the corresponding person-specific gain. The learning-rate parameter determines the slope of the exponential function, whereas the gain parameter determines its height. From the second testing interval onward, as indicated by *I*(*Bn_ti_*>1), the model additionally allows for a within-interval warm-up learning process. Here, *Bn_ti_* denotes the testing-interval number, *r_i_^*^* the person-specific within-interval learning rate, *g_i_^*^* the corresponding gain, and *T_ti_* the testing day within the interval. Rather than estimating the within-interval gain directly, the Bayesian implementation uses a reparameterization to estimate person-specific forgetting, *φ_i_*:

Finally, performance inconsistency is represented by an error term around the exponential trajectory,

where the person-specific standard deviation *σ_i_* quantifies within-person variability in response time.

The model was estimated hierarchically, allowing the process parameters to vary between participants and linking person-specific peak performance, change in peak performance, forgetting, within-person variability, and the within- and across-interval learning rates to age, sex, education, and device type. Age and education were z-standardized.

Peak performance, forgetting, within-person variability, the across-study gain, and both learning-rate parameters were constrained to be positive, whereas change in peak performance was unrestricted. Broad normal priors, N(0,10), were assigned to regression coefficients for peak performance, change in peak performance, forgetting, and the learning-rate parameters. Regression coefficients for within-person variability received N(0,1) priors. Group-level standard deviations received half-normal priors, with scale 10 for all parameters except within-person variability, for which the scale was 1. The parameter constraints and prior specification followed the original implementation.

### 2.2 Bayesian model estimation, convergence diagnostics and model fit

The Bayesian double negative exponential model was estimated in Stan using the *rstan* interface in R. Four Markov chain Monte Carlo chains were run for 6,000 iterations each, including 1,500 warm-up iterations, yielding 18,000 post-warm-up posterior draws for each monitored parameter. Sampling was performed with the No-U-Turn Sampler using adapt_delta = 0.99 and max_treedepth = 15. Convergence was evaluated by visual inspection of trace plots, potential scale reduction factors (R-hat), and effective sample sizes. Diagnostic checks indicated no convergence problems: R-hat values ranged from 0.9998 to 1.0034, no monitored parameter exceeded 1.01, and the minimum effective sample size was 1,330. No divergent transitions occurred after warm-up, and no transitions reached the maximum tree depth. Model fit was quantified using an observation-level coefficient of determination, calculated as

where $\hat{{RT}_{n}}$ denotes the posterior mean person-specific model prediction for observed person-day *n*, and $\bar{RT}$ denotes the mean observed response time across all person-days. The resulting R² was 87%, indicating a good descriptive fit of the BDEM to the observed Face–Name response-time data. Posterior predictive checks showed that the model reproduced the mean trajectories across testing days and intervals, with observed means generally falling within the corresponding 95% posterior predictive intervals. Figure S2 illustrates the posterior predictive check of mean response-time trajectories across testing days and intervals.

## 3. Positron emission tomography imaging acquisition and processing

A subsample of participants underwent amyloid positron emission tomography (PET) with [¹¹C] Pittsburgh Compound B (PiB). Amyloid PET scans were conducted, on average, 0.9 years (± 1.2 years) before the first BRANCH testing interval. Amyloid PET data were available for 198 participants, of whom 167 were classified as amyloid-negative and included in the amyloid-negative sensitivity analysis. Amyloid deposition was quantified using established protocols. PiB images were acquired using a 60-minute dynamic acquisition on either a Siemens ECAT HR+ or a GE PET scanner. Mean PET images were coregistered to T1-weighted magnetic resonance images using SPM12, and the resulting transformation matrices were saved. Regions of interest (ROIs) were defined using FreeSurfer version 6.0 and transformed into PET native space.

To classify participants as amyloid-negative (Aβ–) or amyloid-positive (Aβ+), a global cortical aggregate was calculated based on the mean distribution volume ratio (DVR) across frontal, lateral temporoparietal, and retrosplenial (FLR) regions. Scanner-specific DVR cutoffs were applied to determine amyloid positivity. For the Siemens ECAT HR+ scanner, the FLR DVR cutoff was set at 1.14, consistent with prior work linking this threshold to amyloid accumulation and cognitive decline in clinically unimpaired individuals. For the GE scanner, which has higher sensitivity to amyloid uptake, the corresponding cutoff was set at 1.11.

# Supplementary Figures and Descriptive Information

**Figure S1.** Number of participants contributing data at each BRANCH testing interval.

**Participants who contributed data at testing interval 1**

Total n = 209
(HABS: 119, IADL: 36, SCD = 54)

23 did not contribute interval 3 data

(HABS: 10, IADL: 6, SCD: 7)

35 did not contribute interval 4 data

(HABS: 19, IADL: 5, SCD: 11)

**Participants who contributed data at testing interval 4**

Total n = 73
(HABS: 50, IADL: 14, SCD = 9)

**Participants who contributed data at testing interval 3**

Total n = 108
(HABS: 69, IADL: 19, SCD = 20)

78 did not contribute interval 2 data

(HABS: 40, IADL: 11, SCD: 27)

**Participants who contributed data at testing interval 2**

Total n = 131
(HABS: 79, IADL: 25, SCD = 27)

| **Table S1.** Baseline characteristics by completion status across BRANCH testing intervals. | | | | |
| --- | --- | --- | --- | --- |
| Variable | Overall n = 209 | Completer n = 73 | Non-completer n = 136 | *p-value* |
| Age, M ± SD | 73.6 ± 8.2 | 72.5 ± 8.2 | 74.2 ± 8.4 | 0.162 ^a^ |
| Female sex, n (%) | 136 (65.1) | 53 (72.6) | 83 (61.0) | 0.128 ^b^ |
| Education, M ± SD | 16.5 ± 2.5 | 16.4 ± 2.6 | 16.5 ± 2.5 | 0.777 ^a^ |
| MMSE, M ± SD | 28.9 ± 1.2 | 29.0 ± 1.0 | 28.8 ± 1.3 | 0.148 ^a^ |
| Baseline RT (day 1) ± SD | 3.4 ± 0.9 | 3.3 ± 0.8 | 3.5 ± 0.9 | 0.069 ^a^ |
| Mobile device, n (%) | 95 (45.5) | 30 (41.1) | 65 (47.8) | 0.435 ^b^ |
| Cohort source |  |  |  | 0.005 ^b^ |
| HABS, n (%) | 119 (56.9) | 50 (68.5) | 69 (50.7) |  |
| SCD, n (%) | 54 (25.8) | 9 (12.3) | 45 (33.1) |  |
| IADL, n (%) | 36 (17.2) | 14 (19.2) | 22 (16.2) |  |

*Notes.* ^a^ t-test, ^b^ Pearson’s χ² test (with Yates’ continuity correction for 2x2 tables). Completers were defined as participants who completed all four BRANCH testing intervals; non-completers completed fewer than four intervals.

| **Table S2.** Correct responses and accuracy by testing interval and day. | | | |
| --- | --- | --- | --- |
| Testing interval | Day | Correct responses, M ± SD | Accuracy (%) |
| 1 | 1 | 15.8 ± 3.4 | 39.5 |
| 1 | 2 | 24.7 ± 4.6 | 61.8 |
| 1 | 3 | 29.5 ± 4.3 | 73.8 |
| 1 | 4 | 32.2 ± 4.2 | 80.5 |
| 1 | 5 | 33.1 ± 4.2 | 82.8 |
| 1 | 6 | 34.9 ± 3.4 | 87.3 |
| 1 | 7 | 35.9 ± 3.1 | 89.8 |
| 2 | 1 | 14.8 ± 3.6 | 37.0 |
| 2 | 2 | 25.7 ± 4.3 | 64.3 |
| 2 | 3 | 31.1 ± 4.1 | 77.8 |
| 2 | 4 | 33.3 ± 4.4 | 83.3 |
| 2 | 5 | 34.0 ± 4.4 | 85.0 |
| 2 | 6 | 35.8 ± 3.5 | 89.5 |
| 2 | 7 | 36.2 ± 3.7 | 90.5 |
| 3 | 1 | 14.5 ± 3.3 | 36.3 |
| 3 | 2 | 24.8 ± 4.4 | 62.0 |
| 3 | 3 | 29.4 ± 4.8 | 73.5 |
| 3 | 4 | 32.4 ± 4.3 | 81.0 |
| 3 | 5 | 33.7 ± 4.4 | 84.3 |
| 3 | 6 | 34.7 ± 3.7 | 86.8 |
| 3 | 7 | 34.1 ± 4.8 | 85.3 |
| 4 | 1 | 14.4 ± 3.3 | 36.0 |
| 4 | 2 | 23.3 ± 4.1 | 58.4 |
| 4 | 3 | 28.0 ± 4.6 | 70.0 |
| 4 | 4 | 30.5 ± 4.7 | 76.3 |
| 4 | 5 | 32.9 ± 4.0 | 82.3 |
| 4 | 6 | 33.3 ± 4.7 | 83.3 |
| 4 | 7 | 35.2 ± 3.4 | 88.0 |

**Figure S2.** Posterior predictive check of the Bayesian double negative exponential model. *Points and dashed lines represent the observed means, across participants, of daily median response times for each testing day and interval. Solid lines represent the medians, across posterior predictive draws, of the corresponding model-replicated means, and shaded bands indicate the 95% posterior predictive intervals.*

# Supplementary Sensitivity Analyses

## 4. Amyloid-negative subsample

| **Table S3.** Group-level (population) estimates of Bayesian double negative exponential model (BDEM) parameters for amyloid-negative individuals in the Face-Name Test. \| \| \| \|; Group-level cognitive marker \| *M* \| *SD* \| 2.5% Quantile \| 97.5% Quantile; Peak performance \| 2.06 \| 0.32 \| 2.02 \| 2.11; Change in peak performance \| 0.04 \| 0.08 \| 0.03 \| 0.06; Forgetting between intervals \| 0.97 \| 0.37 \| 0.92 \| 1.03; Short-term learning rate \| 0.70 \| 0.30 \| 0.63 \| 0.77; Long-term learning rate \| 0.63 \| 0.28 \| 0.57 \| 0.69; Within-person variability \| 0.24 \| 0.10 \| 0.23 \| 0.25; *Notes*: SD = standard deviation. M represents the posterior mean of the group-level mean, and SD represents the posterior mean of the group-level between-person standard deviation. The 2.5% and 97.5% quantiles define the 95% credible interval for the group-level mean. \| \| \| \| **Table S4.** Summary of results on associations between cognitive markers and person-level predictors for amyloid-negative individuals in the Face-Name Test. | | | | |
| --- | --- | --- | --- | --- |
| Cognitive marker | Predictor | Mean | 2.5% Quantile | 97.5% Quantile |
| Peak performance | **Age** | **0.16** | **0.10** | **0.23** |
|  | **Sex** | -0.13 | -0.26 | 0.00 |
|  | **Education** | -0.04 | -0.09 | -0.00 |
|  | **Device** | -0.02 | -0.13 | 0.10 |
|  |  |  |  |  |
| Change in peak performance | **Age** | **0.04** | **0.02** | **0.06** |
|  | Sex | -0.02 | -0.07 | 0.03 |
|  | **Education** | **-0.03** | **-0.05** | **-0.01** |
|  | Device | 0.01 | -0.03 | 0.05 |
|  |  |  |  |  |
| Within-person variability | **Age** | **0.04** | **0.02** | **0.06** |
|  | Sex | -0.04 | -0.08 | 0.00 |
|  | Education | -0.01 | -0.02 | 0.01 |
|  | Device | -0.02 | -0.05 | 0.02 |
|  |  |  |  |  |
| Forgetting between intervals | **Age** | **0.13** | **0.04** | **0.21** |
|  | Sex | 0.10 | -0.08 | 0.28 |
|  | Education | -0.02 | -0.10 | 0.05 |
|  | Device | 0.06 | -0.10 | 0.22 |
|  |  |  |  |  |
| Short-term learning rate | Age | -0.08 | -0.16 | 0.01 |
|  | Sex | 0.07 | -0.11 | 0.25 |
|  | Education | -0.04 | -0.11 | 0.04 |
|  | Device | 0.04 | -0.13 | 0.21 |
|  |  |  |  |  |
| Long-term learning rate | **Age** | **-0.16** | **-0.22** | **-0.10** |
|  | **Sex** | **0.13** | **0.02** | **0.25** |
|  | **Education** | **0.06** | **0.01** | **0.11** |
|  | Device | -0.04 | -0.15 | 0.08 |

## 5. Number of testing intervals

### 5.1 Analysis restricted to three testing intervals

| **Table S5.** Group-level (population) estimates of Bayesian double negative exponential model (BDEM) parameters for three testing intervals in the Face-Name Test. \| \| \| \|; Group-level cognitive marker \| *M* \| *SD* \| 2.5% Quantile \| 97.5% Quantile; Peak performance \| 2.12 \| 0.38 \| 2.08 \| 2.16; Change in peak performance \| 0.03 \| 0.05 \| 0.01 \| 0.05; Forgetting between intervals \| 1.05 \| 0.47 \| 0.99 \| 1.11; Short-term learning rate \| 0.75 \| 0.33 \| 0.68 \| 0.83; Long-term learning rate \| 0.66 \| 0.27 \| 0.60 \| 0.72; Within-person variability \| 0.23 \| 0.10 \| 0.22 \| 0.24; *Notes*: SD = standard deviation. M represents the posterior mean of the group-level mean, and SD represents the posterior mean of the group-level between-person standard deviation. The 2.5% and 97.5% quantiles define the 95% credible interval for the group-level mean. \| \| \| \| **Table S6.** Summary of results on associations between cognitive markers and person-level predictors for three testing intervals in the Face-Name Test. | | | | |
| --- | --- | --- | --- | --- |
| Cognitive marker | Predictor | Mean | 2.5% Quantile | 97.5% Quantile |
| Peak performance | **Age** | **0.20** | **0.13** | **0.27** |
|  | **Sex** | **-0.18** | **-0.31** | **-0.05** |
|  | **Education** | **-0.06** | **-0.11** | **-0.01** |
|  | **Device** | 0.05 | -0.06 | 0.17 |
|  |  |  |  |  |
| Change in peak performance | **Age** | **0.02** | **0.01** | **0.03** |
|  | Sex | 0.01 | -0.04 | 0.05 |
|  | **Education** | **-0.02** | **-0.03** | **-0.00** |
|  | Device | 0.01 | -0.03 | 0.04 |
|  |  |  |  |  |
| Within-person variability | **Age** | **0.04** | **0.03** | **0.06** |
|  | **Sex** | **-0.05** | **-0.08** | **-0.01** |
|  | Education | -0.01 | -0.02 | 0.01 |
|  | Device | 0.00 | -0.03 | 0.03 |
|  |  |  |  |  |
| Forgetting between intervals | **Age** | **0.14** | **0.05** | **0.23** |
|  | Sex | 0.07 | -0.12 | 0.26 |
|  | Education | -0.04 | -0.12 | 0.04 |
|  | Device | -0.01 | -0.18 | 0.16 |
|  |  |  |  |  |
| Short-term learning rate | **Age** | **-0.10** | **-0.18** | **-0.01** |
|  | Sex | 0.10 | -0.08 | 0.26 |
|  | Education | -0.03 | -0.11 | 0.05 |
|  | Device | 0.02 | -0.14 | 0.18 |
|  |  |  |  |  |
| Long-term learning rate | **Age** | **-0.15** | **-0.20** | **-0.08** |
|  | **Sex** | **0.14** | **0.02** | **0.25** |
|  | **Education** | **0.06** | **0.01** | **0.12** |
|  | Device | -0.02 | -0.13 | 0.10 |

### 5.2 Analysis restricted to two testing intervals

| **Table S7.** Group-level (population) estimates of Bayesian double negative exponential model (BDEM) parameters for two testing intervals in the Face-Name Test. \| \| \| \|; Group-level cognitive marker \| *M* \| *SD* \| 2.5% Quantile \| 97.5% Quantile; Peak performance \| 2.12 \| 0.35 \| 2.07 \| 2.17; Change in peak performance \| -0.02 \| 0.06 \| -0.04 \| 0.01; Forgetting between intervals \| 1.08 \| 0.52 \| 1.01 \| 1.15; Short-term learning rate \| 0.76 \| 0.33 \| 0.67 \| 0.86; Long-term learning rate \| 0.70 \| 0.31 \| 0.64 \| 0.77; Within-person variability \| 0.22 \| 0.10 \| 0.21 \| 0.23; *Notes*: SD = standard deviation. M represents the posterior mean of the group-level mean, and SD represents the posterior mean of the group-level between-person standard deviation. The 2.5% and 97.5% quantiles define the 95% credible interval for the group-level mean. \| \| \| \| **Table S8.** Summary of results on associations between cognitive markers and person-level predictors for two testing intervals in the Face-Name Test. | | | | |
| --- | --- | --- | --- | --- |
| Cognitive marker | Predictor | Mean | 2.5% Quantile | 97.5% Quantile |
| Peak performance | **Age** | **0.19** | **0.12** | **0.26** |
|  | **Sex** | **-0.16** | **-0.29** | **-0.04** |
|  | **Education** | **-0.06** | **-0.11** | **-0.01** |
|  | **Device** | 0.02 | -0.08 | 0.14 |
|  |  |  |  |  |
| Change in peak performance | Age | 0.02 | -0.02 | 0.05 |
|  | Sex | 0.00 | -0.08 | 0.08 |
|  | Education | -0.01 | -0.03 | 0.02 |
|  | Device | 0.01 | -0.04 | 0.07 |
|  |  |  |  |  |
| Within-person variability | **Age** | **0.04** | **0.03** | **0.06** |
|  | **Sex** | **-0.05** | **-0.08** | **-0.01** |
|  | Education | -0.01 | -0.02 | 0.01 |
|  | Device | 0.01 | -0.02 | 0.04 |
|  |  |  |  |  |
| Forgetting between intervals | **Age** | **0.13** | **0.03** | **0.24** |
|  | Sex | 0.04 | -0.19 | 0.25 |
|  | Education | -0.01 | -0.11 | 0.08 |
|  | Device | -0.02 | -0.22 | 0.17 |
|  |  |  |  |  |
| Short-term learning rate | Age | -0.10 | -0.20 | 0.00 |
|  | Sex | 0.06 | -0.15 | 0.26 |
|  | Education | 0.01 | -0.08 | 0.09 |
|  | Device | 0.01 | -0.17 | 0.19 |
|  |  |  |  |  |
| Long-term learning rate | **Age** | **-0.16** | **-0.22** | **-0.09** |
|  | **Sex** | **0.16** | **0.02** | **0.28** |
|  | **Education** | **0.06** | **0.01** | **0.12** |
|  | Device | -0.04 | -0.17 | 0.09 |

## 6. Task-specific analyses

### 6.1 Face–Name Matching Task

| **Table S9.** Group-level (population) estimates of Bayesian double negative exponential model (BDEM) parameters for the Face-Name Matching Task. \| \| \| \|; Group-level cognitive marker \| *M* \| *SD* \| 2.5% Quantile \| 97.5% Quantile; Peak performance \| 1.74 \| 0.32 \| 1.70 \| 1.78; Change in peak performance \| 0.04 \| 0.07 \| 0.03 \| 0.06; Forgetting between intervals \| 0.98 \| 0.4 \| 0.93 \| 1.03; Short-term learning rate \| 0.84 \| 0.35 \| 0.76 \| 0.92; Long-term learning rate \| 0.64 \| 0.24 \| 0.56 \| 0.72; Within-person variability \| 0.22 \| 0.07 \| 0.21 \| 0.22; *Notes*: SD = standard deviation. M represents the posterior mean of the group-level mean, and SD represents the posterior mean of the group-level between-person standard deviation. The 2.5% and 97.5% quantiles define the 95% credible interval for the group-level mean. \| \| \| \| **Table S10.** Summary of results on associations between cognitive markers and person-level predictors for the Face-Name Matching Task. | | | | |
| --- | --- | --- | --- | --- |
| Cognitive marker | Predictor | Mean | 2.5% Quantile | 97.5% Quantile |
| Peak performance | **Age** | **0.13** | **0.07** | **0.18** |
|  | Sex | -0.09 | -0.21 | 0.03 |
|  | Education | -0.04 | -0.07 | -0.00 |
|  | **Device** | **-0.13** | **-0.23** | **-0.03** |
|  |  |  |  |  |
| Change in peak performance | **Age** | **0.03** | **0.01** | **0.05** |
|  | Sex | -0.01 | -0.05 | 0.03 |
|  | Education | -0.01 | -0.02 | -0.00 |
|  | Device | 0.01 | -0.02 | 0.04 |
|  |  |  |  |  |
| Within-person variability | **Age** | **0.02** | **0.01** | **0.03** |
|  | **Sex** | **-0.04** | **-0.07** | **-0.02** |
|  | Education | 0.00 | -0.01 | 0.01 |
|  | Device | 0.00 | -0.02 | 0.02 |
|  |  |  |  |  |
| Forgetting between intervals | Age | 0.02 | -0.06 | 0.10 |
|  | Sex | 0.05 | -0.11 | 0.22 |
|  | Education | 0.04 | -0.03 | 0.11 |
|  | Device | -0.08 | -0.22 | 0.07 |
|  |  |  |  |  |
| Short-term learning rate | Age | -0.08 | -0.18 | 0.02 |
|  | Sex | 0.10 | -0.10 | 0.28 |
|  | Education | 0.00 | -0.08 | 0.08 |
|  | Device | -0.03 | -0.20 | 0.15 |
|  |  |  |  |  |
| Long-term learning rate | **Age** | **-0.10** | **-0.17** | **-0.03** |
|  | **Sex** | **0.15** | **0.02** | **0.28** |
|  | Education | 0.03 | -0.03 | 0.08 |
|  | Device | 0.01 | -0.11 | 0.14 |

### 6.2 First-Letter Name Recall Task

| **Table S11.** Group-level (population) estimates of Bayesian double negative exponential model (BDEM) parameters for the First-Letter Name Recall Task. \| \| \| \|; Group-level cognitive marker \| *M* \| *SD* \| 2.5% Quantile \| 97.5% Quantile; Peak performance \| 2.49 \| 0.47 \| 2.43 \| 2.56; Change in peak performance \| 0.03 \| 0.06 \| 0.01 \| 0.05; Forgetting between intervals \| 1.14 \| 0.58 \| 1.06 \| 1.22; Short-term learning rate \| 0.7 \| 0.31 \| 0.61 \| 0.8; Long-term learning rate \| 0.72 \| 0.31 \| 0.64 \| 0.81; Within-person variability \| 0.41 \| 0.2 \| 0.39 \| 0.42; *Notes*: SD = standard deviation. M represents the posterior mean of the group-level mean, and SD represents the posterior mean of the group-level between-person standard deviation. The 2.5% and 97.5% quantiles define the 95% credible interval for the group-level mean. \| \| \| \| **Table S12.** Summary of results on associations between cognitive markers and person-level predictors for the First-Letter Name Recall Task. | | | | |
| --- | --- | --- | --- | --- |
| Cognitive marker | Predictor | Mean | 2.5% Quantile | 97.5% Quantile |
| Peak performance | **Age** | **0.27** | **0.18** | **0.35** |
|  | **Sex** | **-0.27** | **-0.44** | **-0.10** |
|  | **Education** | **-0.07** | **-0.13** | **-0.01** |
|  | **Device** | **0.17** | **0.02** | **0.32** |
|  |  |  |  |  |
| Change in peak performance | **Age** | **0.04** | **0.01** | **0.06** |
|  | Sex | 0.02 | -0.03 | 0.07 |
|  | **Education** | **-0.02** | **-0.04** | **-0.01** |
|  | Device | 0.02 | -0.02 | 0.06 |
|  |  |  |  |  |
| Within-person variability | **Age** | **0.08** | **0.05** | **0.11** |
|  | Sex | -0.06 | -0.12 | 0.00 |
|  | Education | -0.02 | -0.05 | 0.01 |
|  | Device | -0.01 | -0.06 | 0.05 |
|  |  |  |  |  |
| Forgetting between intervals | **Age** | **0.23** | **0.12** | **0.34** |
|  | Sex | 0.12 | -0.12 | 0.36 |
|  | Education | -0.09 | -0.19 | 0.01 |
|  | Device | 0.02 | -0.19 | 0.23 |
|  |  |  |  |  |
| Short-term learning rate | Age | -0.03 | -0.13 | 0.06 |
|  | Sex | 0.11 | -0.10 | 0.29 |
|  | Education | -0.03 | -0.11 | 0.06 |
|  | Device | 0.03 | -0.14 | 0.22 |
|  |  |  |  |  |
| Long-term learning rate | **Age** | **-0.17** | **-0.24** | **-0.10** |
|  | Sex | 0.11 | -0.04 | 0.25 |
|  | Education | 0.1 | 0.04 | 0.16 |
|  | Device | -0.07 | -0.2 | 0.07 |

## 7. Completer-only analysis

| **Table S13.** Group-level (population) estimates of Bayesian double negative exponential model (BDEM) parameters from the completer-only analysis restricted to the first three testing intervals of the Face–Name Test. \| \| \| \|; Group-level cognitive marker \| *M* \| *SD* \| 2.5% Quantile \| 97.5% Quantile; Peak performance \| 2.08 \| 0.39 \| 2.03 \| 2.12; Change in peak performance \| 0.03 \| 0.05 \| 0.01 \| 0.05; Forgetting between intervals \| 1.04 \| 0.47 \| 0.99 \| 1.08; Short-term learning rate \| 0.80 \| 0.36 \| 0.73 \| 0.88; Long-term learning rate \| 0.75 \| 0.28 \| 0.66 \| 0.84; Within-person variability \| 0.22 \| 0.09 \| 0.22 \| 0.23; *Notes*: SD = standard deviation. M represents the posterior mean of the group-level mean, and SD represents the posterior mean of the group-level between-person standard deviation. The 2.5% and 97.5% quantiles define the 95% credible interval for the group-level mean. \| \| \| \| **Table S14.** Associations between cognitive markers and person-level predictors in the completer-only analysis restricted to the first three testing intervals of the Face–Name Test. | | | | |
| --- | --- | --- | --- | --- |
| Cognitive marker | Predictor | Mean | 2.5% Quantile | 97.5% Quantile |
| Peak performance | **Age** | **0.18** | **0.09** | **0.27** |
|  | **Sex** | **-0.22** | **-0.40** | **-0.04** |
|  | **Education** | **-0.05** | **-0.10** | **-0.01** |
|  | **Device** | 0.04 | -0.12 | 0.19 |
|  |  |  |  |  |
| Change in peak performance | **Age** | **0.02** | **0.01** | **0.03** |
|  | Sex | 0.00 | -0.05 | 0.05 |
|  | **Education** | **-0.02** | **-0.03** | **-0.00** |
|  | Device | 0.01 | -0.02 | 0.05 |
|  |  |  |  |  |
| Within-person variability | **Age** | **0.03** | **0.01** | **0.06** |
|  | **Sex** | **-0.05** | **-0.09** | **-0.01** |
|  | Education | -0.01 | -0.03 | 0.00 |
|  | Device | 0.01 | -0.03 | 0.05 |
|  |  |  |  |  |
| Forgetting between intervals | **Age** | **0.16** | **0.04** | **0.27** |
|  | Sex | 0.03 | -0.20 | 0.26 |
|  | Education | -0.05 | -0.14 | 0.04 |
|  | Device | -0.01 | -0.20 | 0.20 |
|  |  |  |  |  |
| Short-term learning rate | **Age** | **-0.08** | **-0.20** | **-0.03** |
|  | Sex | 0.17 | -0.05 | 0.38 |
|  | Education | -0.05 | -0.15 | 0.04 |
|  | Device | -0.01 | -0.21 | 0.20 |
|  |  |  |  |  |
| Long-term learning rate | **Age** | **-0.09** | **-0.19** | **-0.01** |
|  | **Sex** | **0.20** | **0.01** | **0.39** |
|  | **Education** | **0.10** | **0.02** | **0.17** |
|  | Device | -0.01 | -0.19 | 0.15 |

## 8. Sensitivity analysis restricted to daily response-time estimates based on at least 15 correct trials

| **Table S15.** Group-level (population) estimates of Bayesian double negative exponential model (BDEM) parameters in the Face–Name Test sensitivity analysis restricted to daily response-time medians based on at least 15 correct trials. \| \| \| \|; Group-level cognitive marker \| *M* \| *SD* \| 2.5% Quantile \| 97.5% Quantile; Peak performance \| 2.07 \| 0.33 \| 2.02 \| 2.11; Change in peak performance \| 0.05 \| 0.09 \| 0.03 \| 0.07; Forgetting between intervals \| 1.11 \| 0.45 \| 1.04 \| 1.18; Short-term learning rate \| 0.73 \| 0.32 \| 0.66 \| 0.80; Long-term learning rate \| 0.63 \| 0.29 \| 0.58 \| 0.70; Within-person variability \| 0.21 \| 0.08 \| 0.20 \| 0.22; *Notes*: SD = standard deviation. M represents the posterior mean of the group-level mean, and SD represents the posterior mean of the group-level between-person standard deviation. The 2.5% and 97.5% quantiles define the 95% credible interval for the group-level mean. \| \| \| \| **Table S16.** Summary of results on associations between cognitive markers and person-level predictors in the sensitivity analysis restricted to daily response-time medians based on at least 15 correct trials. | | | | |
| --- | --- | --- | --- | --- |
| Cognitive marker | Predictor | Mean | 2.5% Quantile | 97.5% Quantile |
| Peak performance | **Age** | **0.17** | **0.11** | **0.23** |
|  | **Sex** | -0.16 | -0.28 | -0.05 |
|  | **Education** | -0.05 | -0.10 | -0.01 |
|  | **Device** | -0.02 | -0.12 | 0.08 |
|  |  |  |  |  |
| Change in peak performance | **Age** | **0.04** | **0.02** | **0.06** |
|  | Sex | 0.01 | -0.04 | 0.06 |
|  | **Education** | **-0.02** | **-0.03** | **-0.01** |
|  | Device | 0.01 | -0.03 | 0.05 |
|  |  |  |  |  |
| Within-person variability | **Age** | **0.03** | **0.02** | **0.05** |
|  | Sex | -0.02 | -0.05 | 0.01 |
|  | Education | -0.00 | -0.02 | 0.01 |
|  | Device | -0.00 | -0.03 | 0.02 |
|  |  |  |  |  |
| Forgetting between intervals | **Age** | **0.12** | **0.03** | **0.22** |
|  | Sex | 0.04 | -0.17 | 0.24 |
|  | Education | -0.00 | -0.08 | 0.08 |
|  | Device | 0.04 | -0.21 | 0.15 |
|  |  |  |  |  |
| Short-term learning rate | Age | -0.08 | -0.17 | 0.00 |
|  | Sex | 0.12 | -0.04 | 0.28 |
|  | Education | -0.01 | -0.08 | 0.07 |
|  | Device | 0.02 | -0.13 | 0.18 |
|  |  |  |  |  |
| Long-term learning rate | **Age** | **-0.16** | **-0.22** | **-0.10** |
|  | **Sex** | **0.12** | **0.01** | **0.23** |
|  | **Education** | **0.06** | **0.01** | **0.11** |
|  | Device | -0.03 | -0.13 | 0.08 |

# References

1.	Oravecz Z, Vandekerckhove J, Hakun JG, Kim SH, Katz MJ, Wang C, et al. Computational Phenotyping of Cognitive Decline With Retest Learning. The Journals of Gerontology: Series B. 2025;80(7).

2.	Oravecz Z, Harrington KD, Hakun JG, Katz MJ, Wang C, Zhaoyang R, et al. Accounting for retest effects in cognitive testing with the Bayesian double exponential model via intensive measurement burst designs. Frontiers in Aging Neuroscience. 2022;14:897343.
